# Case Report: A case of perirectal abscess complicated with rectal necrosis

**DOI:** 10.3389/fsurg.2025.1559084

**Published:** 2025-04-17

**Authors:** Yingfeng Xu, Zhiting Wang, Jianwen Hu, Jiajia Xie, Shiwei Chen, Ke Ke

**Affiliations:** ^1^ Department of Anorectal Surgery, The Seventh Clinical College of Guangzhou University of Chinese Medicine, Shenzhen, China; ^2^ Department of Classical Ward, The Seventh Clinical College of Guangzhou University of Chinese Medicine, Shenzhen, China; ^3^ Department of Traditional Chinese Medicine, Affiliated TCM Hospital of Guangzhou Medical University, Guangzhou, China

**Keywords:** case report, clinical diagnosis and treatment, ischemic proctitis, perirectal abscess, rectal necrosis

## Abstract

Rectal necrosis represents the extreme progression of ischemic proctitis. Given the unique blood supply of the rectum, which features an extensive collateral circulation, cases of rectal necrosis are extremely rare, accounting for 2%–5% of the ischemic colitis cases. The etiology and pathogenesis of rectal necrosis encompass acute vascular occlusion, severe vascular diseases, low blood flow states, and factors such as radiotherapy, vasculitis, and mesenteric venous myointimal hyperplasia. Herein, we report a rare case of perirectal abscess complicated by rectal necrosis at the Anorectal Surgery Department of Bao'an Traditional Chinese Medicine Hospital in Shenzhen. The patient underwent a one-stage incision and drainage for a rectal abscess, followed by sigmoid colostomy and a two-stage reversal of the sigmoid colostomy; all procedures were performed through multidisciplinary collaboration. The surgical outcome was favorable after a 6-month follow-up.

## 1 Introduction

Rectal necrosis may be caused by acute vascular occlusion, severe vascular diseases, or low blood flow states; other causes include radiotherapy, vasculitis, and mesenteric venous myointimal hyperplasia (1). However, the concurrent occurrence of perianal abscess and rectal necrosis is relatively rare in clinical practice but has serious consequences, and there are currently no relevant reports in domestic and foreign literature. We consider that perirectal abscess may affect the blood supply of the rectum through mechanisms such as the spread of local inflammation and vascular compression, thus leading to rectal necrosis. Perirectal abscess complicated by rectal necrosis, though rare, is a critical condition with nonspecific symptoms, presenting significant diagnostic challenges. Traditional diagnostic methods, including digital rectal examination, endoscopy, and imaging, require meticulous interpretation to avoid misdiagnosis. Treatment involves abscess drainage and strategies to enhance rectal blood supply and reduce inflammation. A multidisciplinary approach is crucial for individualized management, improving prognosis and minimizing complications. We report a case managed through abscess drainage and sigmoid colostomy, highlighting the importance of tailored surgical intervention in complex scenarios.

## 2 Case presentation

### 2.1 Clinical data and medical history

In February 2024, a 32-year-old male presented with sudden perianal erythema, burning, mild swelling, tenderness, and incomplete defecation sensation for three days, following prolonged sitting (∼20 h) and consumption of spicy foods. Symptoms worsened during defecation or prolonged sitting, with minimal abnormal stool discharge. The patient denied fever, chills, skin rupture, bleeding, or purulent discharge. Bowel movements were 1–2 times daily with soft stools, accompanied by lower abdominal distension. Due to severe perianal pain, he sought care at our hospital and was diagnosed with “(1) Perianal abscess; (2) Proctitis?” and admitted for surgery. No history of anal trauma, intercourse, or foreign body insertion was reported. In addition, he had no underlying diseases, such as hypertension and diabetes; no history of infectious diseases, trauma, or surgery; no known drug or food allergies; and no history of hereditary diseases.

Physical Examination: The patient was in the lithotomy position. Perianal skin showed no erosion or excrescence. At the 7 o'clock position, 3 cm from the anus, a 3 × 2 × 2 cm oval-shaped elevation with localized redness and swelling was noted. At the 11 o'clock position, a 1 × 1 × 0.5 cm mass with normal overlying skin was observed. Digital examination revealed elevated temperature and significant swelling in the middle and lower rectum. The 7 o'clock mass was tender, with mild tenderness at the anal sinus; slight fluctuation was felt, but no pus was extruded. The 11 o'clock mass was non-tender.

Laboratory Findings on the Day of Admission: Infection indicators showed significant elevations, including the white blood cell count (14.99 × 10^9^/L), neutrophil count (12.75 × 10^9^/L), high-sensitivity C-reactive protein (145.35 mg/L), and procalcitonin (22.35 ng/ml). Liver and kidney function tests revealed hyperglycemia (21.41 mmol/L), elevated total bilirubin (63.0 μmol/L), and decreased carbon dioxide levels (14.0 mmol/L). Blood gas analysis indicated a decreased partial pressure of carbon dioxide (33.0 mmHg) and total carbon dioxide (21.4 mmol/L), along with elevated whole blood lactic acid (3.300 mmol/L). The fingertip blood ketone level was measured at 1.0 mmol/L.

Subsequently, the patient's C-peptide level was measured (0.43 ng/ml; reference range: 1.10–4.40), and consultations were requested from the Departments of Critical Care Medicine and Endocrinology at our hospital. The patient currently presents with symptoms of dry mouth and polydipsia, but no signs of polyuria, limb weakness, or significant weight loss have been observed. There is no prior history of diabetes, although a family history of diabetes is noted. Based on the clinical presentation, the patient is preliminarily diagnosed with ketoacidosis induced by stress hyperglycemia, and the possibility of diabetes cannot be ruled out. Symptomatic treatment, including blood glucose reduction and fluid supplementation for ketone elimination, has been initiated temporarily.

**Figure 1:**
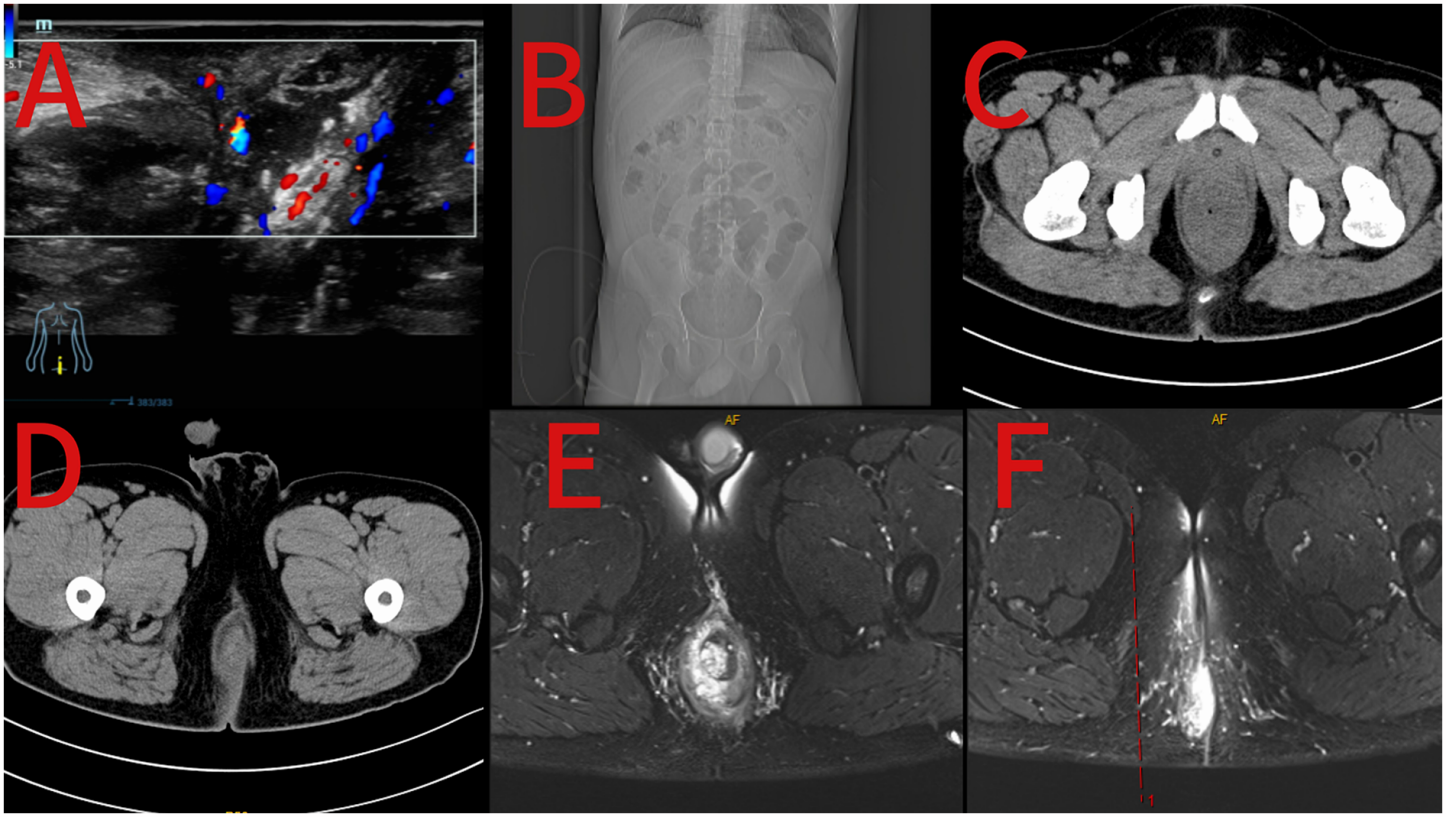
Preoperative ultrasound image of the perianal mass **(A)**; preoperative coronal view of abdominal CT **(B)**; preoperative axial view of abdominal CT **(C,D)**; preoperative 3T MRI images of the anus **(E,F)**.

On the second day of admission, the electrocardiogram showed a heart rate of 140 beats per minute, and the blood pressure was 111/85 mmHg. Fever was present, with a body temperature of 39.9°C. Laboratory tests revealed electrolyte disturbances, including hypokalemia (2.95 mmol/L). No significant abnormalities were observed in other parameters, such as erythrocyte sedimentation rate, coagulation function, blood type, blood lipids, or infectious disease screening. Color Doppler ultrasound of the superficial mass (Figure 1) indicates a mixed mass on the right side of the anus, and its nature remains undetermined. Thoracoabdominal CT (Figure 1) suggests peritonitis with surrounding exudation and fluid accumulation, as well as thickening of the rectal shape. 3T anorectal MRI (Figure 1) shows edema of the rectal and sigmoid colon walls; an abnormal signal below the anal canal, which is considered to be the formation of hemorrhoids; edema of the perirectal fat space and the subcutaneous fat layer of the right buttock; and peritonitis is suspected.

### 2.2 Treatment-related situations

#### 2.2.1 Preoperative analysis

Based on the patient's medical history, physical examination findings, and auxiliary test results, the following diagnoses were made: (1) Perianal abscess; (2) Perirectal abscess; (3) Sepsis/Septic shock; (4) Electrolyte disorder; (5) Proctitis (?); and (6) Urinary retention. The infection had spread, showing a tendency to involve surrounding areas, with signs of peritonitis and hyperglycemia. The possibility of necrotizing fasciitis could not be excluded. Patients with perianal abscesses that are limited to the deep intersphincteric space and infection spreading to the perirectal area, who may present with necrotizing fasciitis and a combined state of hyperglycemia, after excluding surgical contraindications, should be immediately treated as follows: “perianal abscess incision and drainage, perirectal abscess incision and drainage, and perianal/rectal abscess cavity curettage (with preparation for complex perianal necrotizing fasciitis debridement)”.

#### 2.2.2 Findings during the first surgery

A puncture was performed on the skin elevation at the 7 o'clock position of the perianal area, and a very small amount of purulent and bloody secretions was observed. The sphincter space was dissected 3 cm away from the anal verge at the 7 o'clock position, and no necrotic tissue or purulent secretions were found. Exploration along the sphincter space up to the deep and horizontal 4 o'clock position revealed no obvious abnormalities. Anoscopic examination showed that the rectal mucosa was swollen, with scattered dark red ecchymoses, no bleeding points, and a small amount of purulent and bloody secretions. Therefore, the operation was changed to “perianal abscess incision and drainage+perirectal abscess incision and drainage”. The surgical procedure was smooth, the intraoperative blood loss was approximately 10 ml, and the patient was safely returned to the ward.

#### 2.2.3 Evaluation and the second surgery

Following the initial operation, the patient remained sedated. Vital signs included a heart rate of 127 bpm, respiratory rate of 18 breaths/min, SPO2 of 89%, and BP of 71/40 mmHg. Procalcitonin was elevated (55.62 ng/ml). Blood gas analysis revealed metabolic acidosis: pH 7.286, lactate 2.000 mmol/L, H + 51.80 mmol/L, base excess −10.1 mmol/L, buffer base 37.80 mmol/L, and total CO2 16.0 mmol/L. Based on clinical deterioration, laboratory results, and intraoperative findings, septic shock was suspected, and necrotizing fasciitis could not be excluded.

The patient was transferred to the ICU for further management. Tracheal intubation and mechanical ventilation were initiated. Broad-spectrum antibiotics (imipenem-cilastatin, clindamycin, vancomycin) were administered. A central venous catheter was placed, and norepinephrine infusion was started for BP support. Fluid resuscitation with lactated Ringer's solution and human serum albumin was provided to improve colloid osmotic pressure. Sedation, analgesia, and continuous vital sign monitoring were implemented.

**Figure 2:**
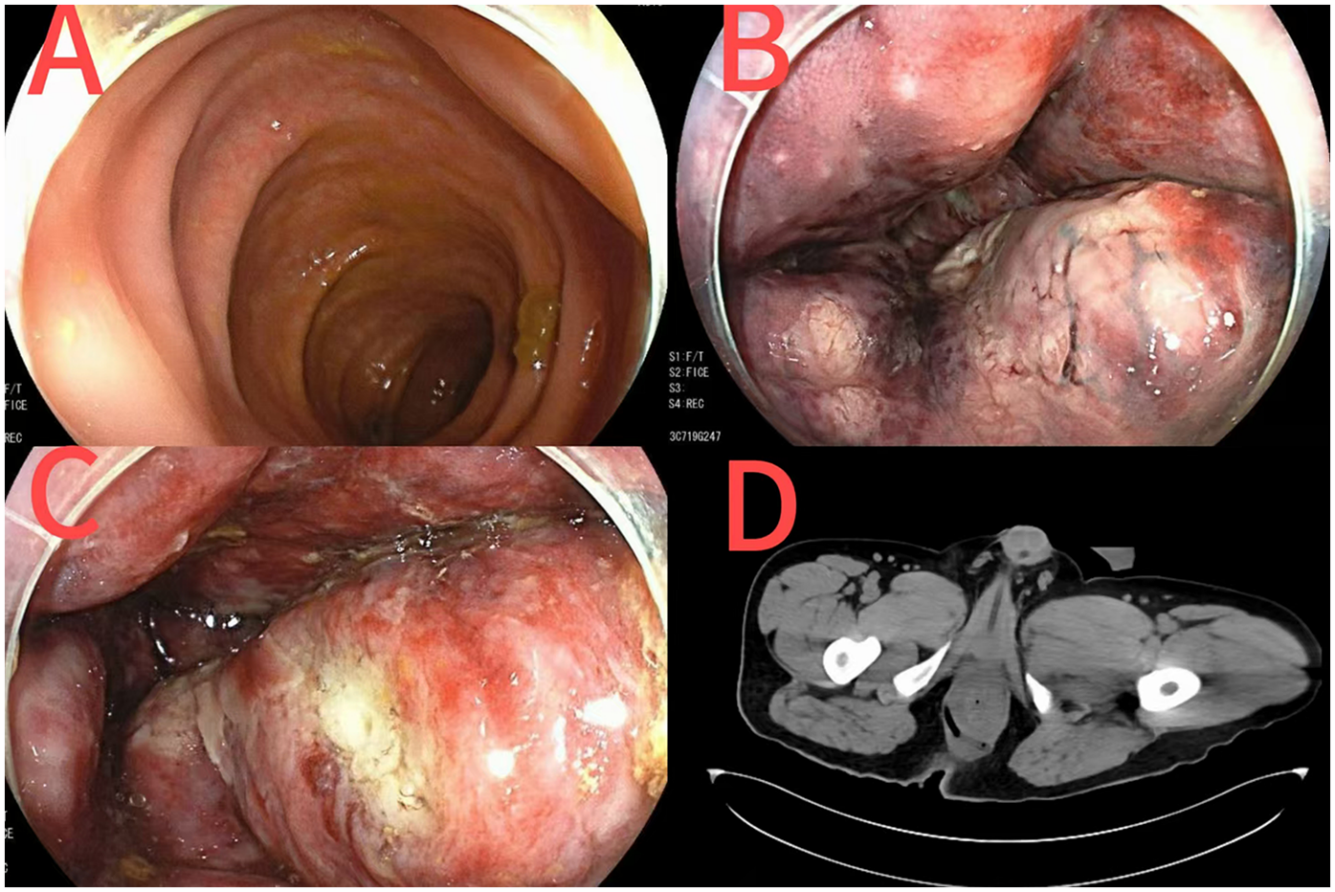
Colonoscopy images after perianal abscess surgery [**(A)** sigmoid colon; **(B)** the vascular network of the rectum turns dark red; **(C)** rectal ulcer]; CT images of the anorectum after perianal abscess surgery **(D)**.

On postoperative day 1, colonoscopy was performed due to rectal morphological changes and ischemic-like mucosal alterations observed during surgery. Findings suggested proctitis (ischemic or infectious) (Figure 2). On day 2, the patient's vital signs remained unstable. Consultations with anorectal, general surgery, and critical care teams were requested. Abdominal CT (Figure 2) revealed rectal and sigmoid colon wall thickening and edema, with mild mucosal and muscular layer enhancement in the lower rectum. Inflammatory changes were noted in the lower abdomen and pelvis, accompanied by fluid accumulation and bilateral pleural effusion. A linear tubular shadow was observed near the right posterior anal canal.

Following the initial surgical intervention, the patient's clinical symptoms showed no significant improvement, with unstable vital signs and progression to septic shock. Despite aggressive conservative management, the condition remained refractory. Based on a comprehensive analysis of auxiliary examinations, concurrent rectal necrosis involving the muscular layer was strongly suspected, necessitating surgical intervention. After communicating with the patient's family, he was sent to the operating room for “laparoscopic exploration+lysis of abdominal adhesions+peritoneal lavage and drainage+sigmoid colostomy” under general anesthesia with endotracheal intubation.

**Figure 3:**
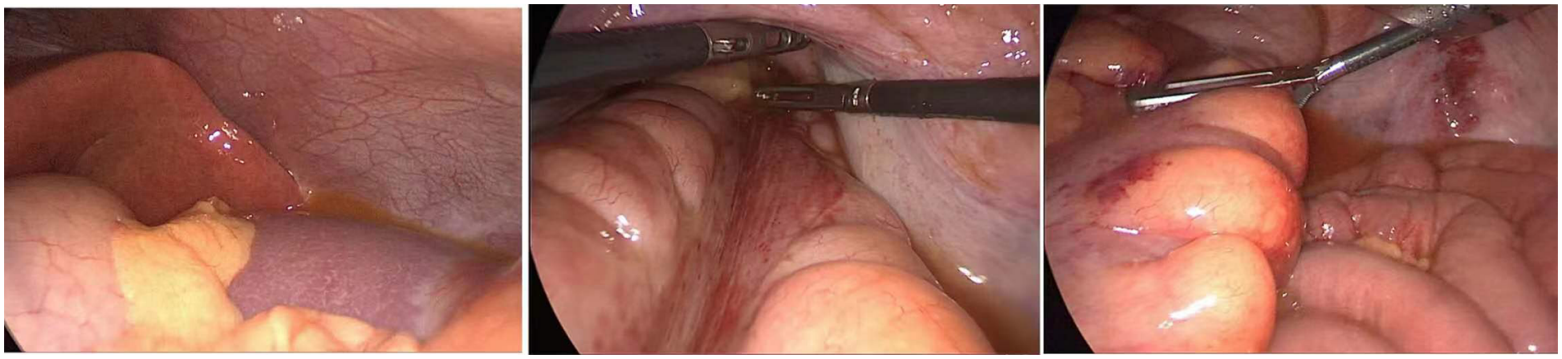
Findings of laparoscopic exploration during the second surgery.

During the exploration, large amounts of brown purulent secretions were found in the abdominal cavity from the splenic fossa to the pelvic cavity (Figure 3). No additional abnormalities were noted during the remaining exploration. Adhesions in the sigmoid and descending colon were lysed. The left pararectal space was dissected along the rectal lateral wall, revealing yellow purulent exudate; no significant pus was observed on the right side. Postoperatively, the patient was transferred to the ICU with drainage tubes for further management.

#### 2.2.4 Postoperative management

Postoperatively, the patient remained sedated and intubated. Management included fasting, gastrointestinal decompression, prokinetics, and potassium supplementation to prevent ileus, alongside continuous anti-infection therapy, prophylactic anticoagulation, antipyretics, and gastrointestinal decompression. By postoperative day 2, the sigmoid colostomy stoma showed gas passage. On day 5, the patient was alert with stable hemodynamics, declining fever, and improving infection markers, prompting transfer to the general ward for continued anti-infection therapy, blood glucose control, and wound care.

By hospital day 18, the patient stabilized with controlled blood glucose, tolerance to a liquid diet, and a functional stoma allowing gas and stool passage without abdominal pain or distension. Physical examination revealed no significant cardiopulmonary or abdominal abnormalities, and the surgical site showed no bleeding or exudation. Routine urine, stool, and blood biochemical markers improved. The patient was discharged with a planned stoma reversal scheduled for three months later.

### 2.3 Readmission

Over three months postoperatively, the patient returned for sigmoid colostomy closure in good general condition. The left lower abdominal stoma showed no redness, swelling, or retraction, with normal blood supply and active fecal discharge. Abdominal surgical scars were well-healed.

Fasting blood glucose was normal. C-peptide levels (fasting, 1–2 h) were 1.92 ng/ml, 4.64 ng/ml, and 2.44 ng/ml, respectively (reference: 1.10–4.40 ng/ml).

**Figure 4:**
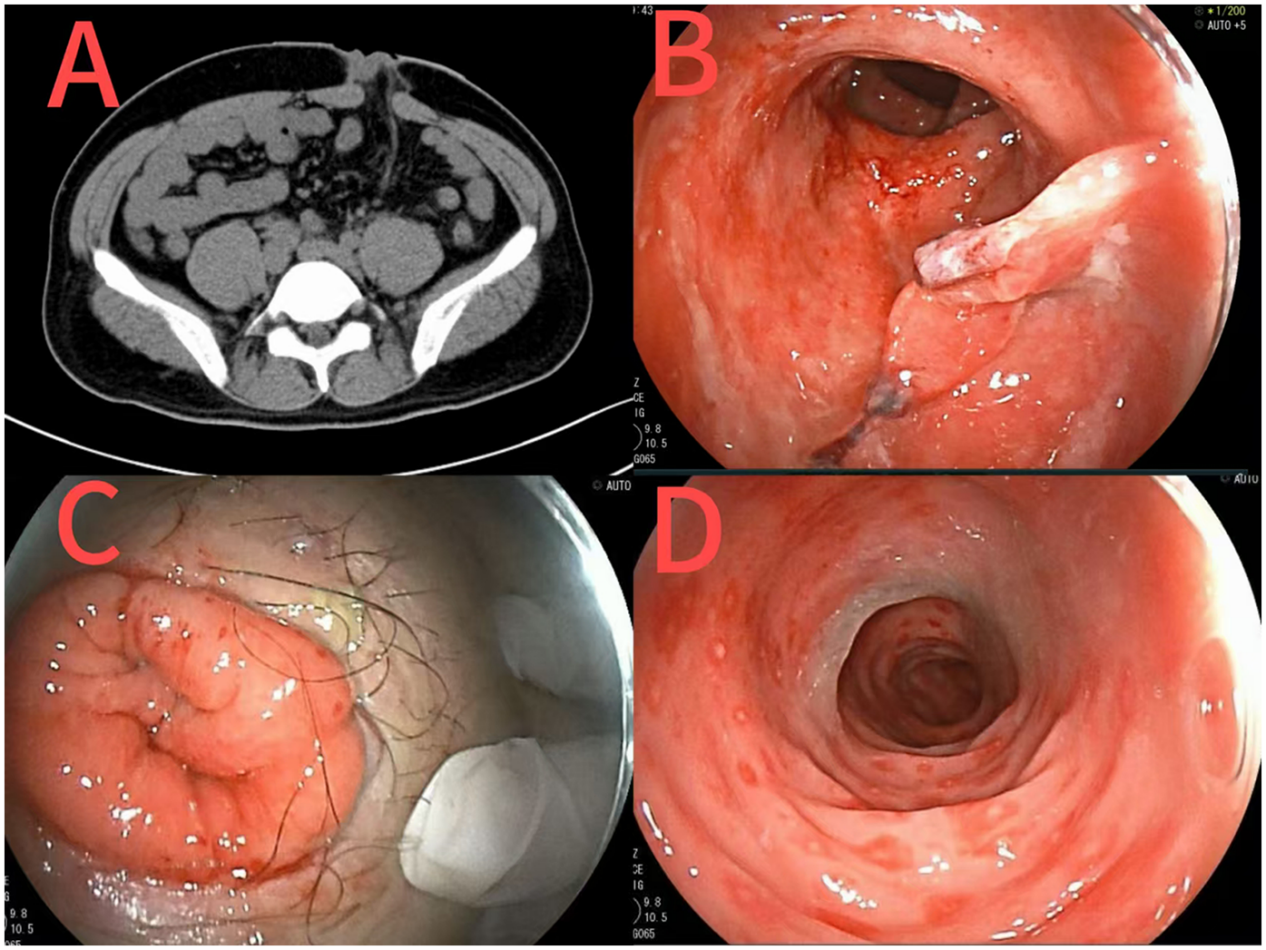
CT images after sigmoid colostomy at the second admission **(A)**; Images of changes after rectal surgery at the second admission [**(B)** rectal stoma; **(C)** abdominal stoma; **(D)** sigmoid colon].

Abdominal and chest CT revealed post-colostomy changes and resolved abdominal/pelvic inflammation (Figure 4). Colonoscopy (Figure 4) showed erosive rectal lesions (suspected inflammatory bowel disease, pending pathology), rectal protuberance, and post-resection changes.

On hospital day 3, after excluding surgical contraindications, the patient underwent laparoscopic sigmoid colostomy reversal and adhesiolysis under general anesthesia. The procedure was successful, with no postoperative complications. By day 12, normal anal function (gas and stool passage) was restored, and the patient was discharged with satisfactory recovery.

### 2.4 Follow-up

A telephone follow-up was conducted within one year postoperatively. The patient reported no significant discomfort but noted slightly less smooth defecation compared to before. No notable perianal discomfort was observed. The patient was not taking hypoglycemic drugs, and blood glucose levels remained within the normal range. He was advised to undergo regular follow-up examinations, including electronic gastroenteroscopy, and to seek medical attention if any discomfort arose.

## 3 Discussion

The rectum, located at the distal end of the digestive tract, benefits from a robust vascular network and extensive collateral circulation. Its primary blood supply originates from the superior rectal artery, a branch of the inferior mesenteric artery, supplemented by the middle and inferior rectal arteries, which arise from the internal iliac and internal pudendal arteries, respectively. Additional collateral circulations exist between the lumbar and internal iliac arteries, along with extensive intramural anastomoses, particularly in the distal rectum. This vascular redundancy ensures rectal viability even with partial supply compromise, making rectal necrosis exceedingly rare. Only isolated case reports exist, with no established incidence data (2). As a rare and severe complication of perianal abscess, rectal necrosis remains an exceptionally uncommon clinical entity.

Current evidence suggests that rectal necrosis may be associated with severe atherosclerosis or cardiovascular disease leading to significant hemodynamic instability. For instance, Yunlong et al. reported a case of idiopathic intestinal necrosis in a patient with coronary atherosclerotic heart disease (3). Similarly, a study documented primary rectal necrosis following cardiopulmonary resuscitation in a patient with a history of coronary heart disease and diabetes (4). Tomoya Iida's team described rectal necrosis secondary to abdominal aortic aneurysm rupture (1). Additionally, Maun et al. and Azimuddin reported cases of severe rectal necrosis in patients with underlying cardiovascular conditions, which compromised hemodynamic stability (2, 5). Other contributing factors include chronic constipation, use of medications with constipating side effects, severe adverse reactions to sodium phosphate enemas, and systemic conditions such as lupus-associated vasculitis, all of which have been implicated in the pathogenesis of rectal necrosis (6–8).

Anatomically, the colonic vasculature exhibits vulnerable zones at Sudeck's and Griffith's points. Sudeck's point, located at the splenic flexure, represents the anastomosis between the marginal vascular arches of the transverse and descending colon. Studies indicate that approximately 50% of cases demonstrate inadequate anastomosis at this site, rendering the splenic flexure a frequent location for ischemic colitis (9). Griffith's point, situated at the junction of the sigmoid artery's lowest branch and the superior rectal artery, is termed Sudeck's critical area and is another common site for ischemic bowel disease (10).

Based on the patient's symptoms, signs, and auxiliary examinations, an initial diagnosis of perianal abscess or associated proctitis was made. After admission, the condition rapidly progressed to sepsis with metabolic disturbances, and CT revealed peritonitis. The case was deemed to have evolved from a perirectal abscess to necrotizing fasciitis, likely causing peritoneal effusion. Emergency surgery was performed for lesion debridement and intensive anti-infective therapy. Initial intersphincteric abscess drainage yielded minimal pus, with imaging suggesting perirectal inflammation spread. Postoperatively, symptoms and laboratory parameters showed no improvement. Further evaluation confirmed lower rectal necrosis extending to the muscular layer, prompting laparoscopic sigmoid double-barrel colostomy, after which the patient's vital signs stabilized.

Rectal necrosis is typically attributed to vascular, intestinal, or anastomotic weak zone factors. In this case, cardiovascular diseases were ruled out via lipid profile, arterial Doppler ultrasound, and cardiac ultrasound. No history of enema or chronic constipation was noted, and necrosis occurred in the lower rectum, not the weak zones. Therefore, we propose the following mechanisms for the patient's rectal necrosis:

First, the patient had a history of prolonged sitting before the onset of symptoms, which may have caused local circulatory obstruction due to sustained pressure on the buttocks, potentially triggering the development of a perianal abscess. Second, the infection from the perianal abscess was not effectively controlled, allowing bacteria to potentially spread to the rectal wall through blood vessels or lymphatic channels, leading to tissue infection and ischemic necrosis. Alternatively, bacterial toxins may have damaged vascular endothelial cells, impairing blood circulation and resulting in tissue necrosis. Finally, the inflammatory response from the perianal abscess may have elevated local tissue pressure, compressing perirectal blood vessels and compromising rectal blood supply.

Considering the patient's rapid clinical deterioration after admission, lack of symptom improvement following perianal abscess drainage, concurrent septic shock, and endoscopic findings of ischemic changes in the rectum, we conclude that the rectal necrosis was secondary to severe infection spread from the perianal abscess and circulatory obstruction due to prolonged sitting, ultimately leading to ischemic proctitis and rectal necrosis.

Regarding the patient's hyperglycemic state, although C-peptide levels during both hospital admissions indicated impaired pancreatic islet function, blood glucose levels normalized postoperatively and during follow-up. Studies have shown that severe physiological stress (e.g., sepsis) can induce metabolic abnormalities (e.g., elevated cortisol, glucagon, and catecholamines), leading to stress-induced hyperglycemia (11). Therefore, we attribute the patient's hyperglycemia to stress-induced hyperglycemia secondary to sepsis rather than an underlying diabetic condition.

Rectal necrosis is categorized into non-gangrenous and gangrenous types. Non-gangrenous necrosis, a transient condition limited to the mucosa and submucosa, presents with edema, submucosal hemorrhage, and partial mucosal necrosis, often leading to mucosal sloughing and ulceration. Typical features include multiple superficial or confluent ulcers, indicative of acute non-hemorrhagic necrosis (12). In contrast, gangrenous necrosis involves transmural damage, potentially progressing to perforation or hemorrhage (12).

The diagnosis of rectal necrosis requires a combination of clinical signs, symptoms, and auxiliary examinations. Typical symptoms of ischemic colitis include abdominal pain, distension, diarrhea, hematochezia, nausea, vomiting, and fever, though these are non-specific. Physical signs may include peritoneal irritation and diminished bowel sounds (13, 14). Imaging and endoscopic examinations play crucial roles in diagnosis. CT scans may reveal bowel wall thickening, edema, perirectal effusion, or pneumatosis intestinalis in ischemic segments, although early-stage lesions may appear normal (2, 14, 15). According to ACG guidelines, endoscopy is the primary diagnostic technique, with biopsy performed if necessary. Patients suspected of intestinal necrosis should undergo colonoscopy as early as possible, which also aids in differentiating conditions such as Crohn's disease and ulcerative colitis (10, 12–14).

Treatment options for rectal necrosis include non-surgical and surgical approaches. Conservative management, effective for submucosal necrosis, involves targeted antibiotic therapy based on culture and sensitivity testing, administered for at least 72 h as recommended by ACG (10), alongside fluid and electrolyte balance and nutritional support to enhance resistance and hemodynamic stability. For perirectal abscess with rectal necrosis, surgical intervention begins with abscess drainage and necrotic tissue removal, followed by diversion surgery or rectal resection based on necrosis extent. In this case, the patient underwent abscess drainage and sigmoid colostomy, with successful stoma reversal after three months. Diversion surgery is preferred initially to restore rectal function, with stoma reversal planned later; complete necrosis may require partial or total rectal resection to prevent sepsis (5). Non-gangrenous necrosis can be managed conservatively, while gangrenous necrosis, due to perforation and hemorrhage risks, necessitates surgery (12).

## 4 Conclusion

We report a case of perirectal abscess complicated by rectal necrosis. Prompt diagnosis, surgical debridement of necrotic tissue, and systemic antimicrobial therapy led to successful recovery. This case highlights the importance of considering rectal necrosis in rapidly progressive perirectal abscesses unresponsive to conventional drainage. Treatment strategies should be dynamically tailored based on diagnostic findings and clinical progression. Our findings underscore the need for increased clinical vigilance regarding this rare but critical condition and offer valuable insights into managing rectal necrosis secondary to perirectal abscess.

## Data Availability

The original contributions presented in the study are included in the article/Supplementary Material, further inquiries can be directed to the corresponding author.
